# Dengue seroprevalence in a cohort of schoolchildren and their siblings in Yucatan, Mexico (2015-2016)

**DOI:** 10.1371/journal.pntd.0006748

**Published:** 2018-11-21

**Authors:** Norma Pavía-Ruz, Gloria Abigail Barrera-Fuentes, Salha Villanueva-Jorge, Azael Che-Mendoza, Julio César Campuzano-Rincón, Pablo Manrique-Saide, Diana Patricia Rojas, Gonzalo M. Vazquez-Prokopec, M. Elizabeth Halloran, Ira M. Longini, Héctor Gómez-Dantés

**Affiliations:** 1 Centro de Investigaciones Regionales “Dr. Hideyo Noguchi”, Universidad Autónoma de Yucatán, Merida, Yucatan, Mexico; 2 Laboratorio Estatal de Salud Pública y Referencia Epidemiológica, Servicios de Salud de Yucatán, Merida, Yucatan, Mexico; 3 Centro de Investigación en Salud Poblacional, Instituto Nacional de Salud Pública, Cuernavaca, Morelos, Mexico; 4 Campus de Ciencias Biológicas y Agropecuarias, Universidad Autónoma de Yucatán, Merida, Yucatan, Mexico; 5 Department of Biostatistics, University of Florida, Gainesville, Florida, United States of America; 6 Center for Inference and Dynamics of Infectious Diseases, Seattle, Washington, United States of America; 7 Department of Environmental Sciences, Emory University, Atlanta, Georgia, United States of America; 8 Vaccine and Infectious Disease Division, Fred Hutchinson Cancer Research Center, Seattle, Washington, United States of America; 9 Department of Biostatistics, University of Washington, Seattle, Washington, United States of America; 10 Center for Health Systems Research, National Institute of Public Health, Mexico City, Mexico; University of Washington, UNITED STATES

## Abstract

**Background:**

The implementation of vector control interventions and potential introduction new tools requires baseline data to evaluate their direct and indirect effects. The objective of the study is to present the seroprevalence of dengue infection in a cohort of children 0 to 15 years old followed during 2015 to 2016, the risk factors and the role of enhanced surveillance strategies in three urban sites (Merida, Ticul and Progreso) in Yucatan, Mexico.

**Methods:**

A cohort of school children and their family members was randomly selected in three urban areas with different demographic, social conditions and levels of transmission. We included results from 1,844 children aged 0 to 15 years. Serum samples were tested for IgG, NS1 and IgM. Enhanced surveillance strategies were established in schools (absenteeism) and cohort families (toll-free number).

**Results:**

Seroprevalence in children 0 to 15 years old was 46.8 (CI 95% 44.1–49.6) with no difference by sex except in Ticul. Prevalence increased with age and was significantly lower in 0 to 5 years old (26.9%, 95% CI:18.4–35.4) compared with 6 to 8 years old (43.9%, 95% CI:40.1–47.7) and 9 to 15 years old (61.4%, 95% CI:58.0–64.8). Sharing the domestic space with other families increased the risk 1.7 times over the individual families that own or rented their house, while risk was significantly higher when kitchen and bathroom were outside. Complete protection with screens in doors and windows decreased risk of infection. Seroprevalence was significantly higher in the medium and high risk areas.

**Conclusions:**

The prevalence of antibodies in children 0 to 15 years in three urban settings in the state of Yucatan describe the high exposure and the heterogenous transmission of dengue virus by risk areas and between schools in the study sites. The enhanced surveillance strategy was useful to improve detection of dengue cases with the coincident transmission of chikungunya and Zika viruses.

## Author summary

Dengue is a major public health problem in Latin America. Its transmission is highly heterogeneous, and its burden varies by geographic region, age group affected, serotype and other factors. While surveillance of dengue in the region has improved, several limitations remain, including under detection, misdiagnosis and the complexity of controlling a vector that has adapted to human dwellings in tropical and subtropical urban contexts. Prospective studies have become crucial to understand the transmission of dengue in urban environments and assess the impact of control strategies, such as the introduction of a dengue vaccine or additional vector control interventions. Our findings provide epidemiological data regarding the serological profile and risk factors for dengue infections in a cohort of children 0 to 15 years old in an endemic state in Mexico and confirmed the high exposure in these age groups. Likewise, enhanced and passive surveillance of cases gave us the opportunity to measure the behavior of dengue activity during chikungunya and Zika viruses’ arrival, which we believe will contribute to improve the design of surveillance and control strategies.

## Introduction

Dengue is a major public health problem in Latin America due to the increasing trend of cases, the vast urban areas affected, and the complexity of controlling a vector that has adapted to human dwellings in tropical and subtropical urban contexts [1]. Accurate estimates of the burden of dengue [2] are difficult because of the high proportion of asymptomatic infections, the syndromic nature of the clinical spectrum that allows for misdiagnosis with other viral infections [3], the limited capacities of the surveillance systems, and the low demand for health services by affected populations [4–6]. Transmission of the four dengue serotypes in endemic countries is heterogeneous with respect to the age groups affected, the seasonality, and the intensity and severity of epidemics[7].

An improved understanding of the complex dynamic of factors involved in dengue transmission requires the characterization of different parameters related to the incidence of asymptomatic, sub-clinical and symptomatic infections [8,9]; the prevalence and seroconversion rates by age group and sex; the herd immunity to specific serotypes [10]; the profile of primary and secondary infections and risk factors associated with severe dengue; as well as their relationship with the entomological variables at the individual, household, neighborhood, locality and regional levels [11–14].

Prospective studies have become crucial for understanding dengue transmission in urban settings and are invaluable in providing the data required to effectively evaluate the impact of traditional and innovative control strategies [15,16]. In endemic areas, transmission dynamics can be better understood with the longitudinal study of young and naïve populations [17]. Selecting school children as the basis for a cohort has several advantages. They are generally susceptible to dengue infection; can be involved and followed-up for longer periods through their attendance to nearby schools; their families are responsive and supportive to health initiatives arising from the educational institutions, and their households are near the school facilitating logistics for follow-up and allowing for a febrile and absentee school-based surveillance system [18] to monitor under reporting of febrile and dengue cases. In addition, there are concerns about the specific risks that school grounds may have in triggering transmission in certain environments [19].

The availability of a licensed vaccine poses different challenges to current dengue control programs since its gradual introduction is not expected to cover all susceptible and at-risk populations or even provide complete immunity in target groups. In each of these populations there are several questions that need to be addressed regarding the clinical spectrum and transmission risks where the vaccine or any other control innovation may provide a potential benefit [20].

The objective was to describe the seroprevalence of dengue infection in a cohort of children from elementary and middle schools in three urban sites with different socio-demographic-economic profiles and transmission patterns in the state of Yucatan, Mexico. This study presents the seroprevalence status, the socio-demographic risk factors associated with dengue infection, and the results of enhanced surveillance strategies established to support detection of dengue cases from 2015 through 2016.

## Methods

This cohort study was designed to generate baseline epidemiological information of dengue transmission during 2015 and 2016 in three urban areas in the southern state of Yucatan, Mexico.

### Study sites

Merida is the capital and major human settlement of Yucatan State, with 892,363 inhabitants (42.5% of the state population) and 257,826 (46.4%) houses. It is the most important economic city, concentrating 50% of the industrial activity. Approximately 1 million national and 250,000 international tourists visit Merida every year [21]. Climate in Yucatan is warm and humid, and the rainy season falls between June and October; the mean annual temperature is 25.9◦C (19.5 to 33.6) and annual precipitation is 1050 (mm). Merida concentrates ~60% of all dengue cases reported in the state. Ticul is an urban locality, located 96 kilometers south of Merida with 40,161 people and about 9,808 houses, concentrates around 3% of all dengue cases in the state. Progreso is the major seaport located 27 km north of Merida. It has 59,122 people and about 16,020 houses. Progreso is the most popular beach resort and tourist destination for many local citizens as well as national and international visitors (291,709 tourists and 136 cruises ship every year). Consequently, most inhabitants of Progreso (60.4%) are involved in commercial and tourist services. Progreso represents around 1% of all dengue cases reported in Yucatan.

The cohort study was initially defined by a random selection of five extensive geographical areas within those cities. These included: two low risk areas (one urban area in the north of Merida and the town of Progreso); two medium risk areas (one urban area in central Merida and the town of Ticul); and one high-risk urban area in the south of Merida. The definition of risk was determined by the historical reports of the number of dengue cases, the percent of cases reported every year and the continuous transmission during 6 to 8 or more weeks every year provided by the state surveillance system [22].

The entire study population was composed of a cohort of children (index children) in elementary and middle schools together with their family members sharing the same home with the index children (family equals number of index children). This report describes the results from the cohort of index school children and their siblings up to 15 years of age.

### Enrolment of school children and their families

The State Ministry of Education of Yucatan provided a list of elementary public schools located in the selected areas, and a cohort of children from 1^st^ to 3^rd^ grade (6 to 8 years old) was randomly selected from eight schools in Merida, four in Progreso and two from Ticul. A convenience sample of 50 children per grade was defined to gather 150 school children (index children) in each of the five risk areas (450 for Merida (150 in each low, middle high risk areas), 150 for Ticul (middle risk) and 150 for Progreso (low risk)). Because children from different grades could come from the same family, we restricted the selection to one child per family to have 50 different and independent families per grade. Recruitment of school-aged children (between 6 to 12 years) included invitation to all other members of the household of the children enrolled. Consent and assent forms were obtained individually from each adult and from parents in the case of children and participants younger than 18 years old and were signed before blood samples were taken. Exclusion criteria included refusal to participate or plans to move outside of the study area during the months following enrolment.

The enrolment of new school children in the 2^nd^ year (2016) was designed to incorporate 50 new 1^st^ grade children per risk area from the same elementary schools or additional ones when required. Based on the results of the dengue vaccine trials [23] the target groups for the vaccine were children over 9 years old instead of under-five years old. Therefore, the recruitment scheme changed to include more children aged 9 to 12 years old from new middle schools. From the additional 150 new children required from middle schools in Merida, only 134 were recruited along with 37 from elementary schools (171 new index children). Ticul and Progreso recruited 50 middle school children each plus 19 and 16 new index children from elementary schools, respectively.

Baseline and follow-up evaluations of the cohort population were obtained during the period December 2014 through August 2016 with baseline demographic information, clinical history of dengue and blood samples taken for serological evidence of dengue virus (DENV) infection after the annual transmission season (August to December). Baseline and first follow up evaluation are presented here.

### Individual, family and household questionnaires

The data collected included individual, family and household questionnaires and was obtained by a multidisciplinary field team called “Familias sin Dengue” (FSD = Families without dengue) integrated by physicians, nurses, social scientists and technical personnel. Basic data regarding house characteristics included construction material, number of rooms, sanitation services (potable water, sewage, garbage collection), water use patterns, and physical protection of windows such as screens in doors and windows. The individual and family questionnaires included basic demographic data (age, sex, education level, occupation, among others) together with a clinical history of dengue including signs and symptoms, dates of occurrence, access or utilization of health services and hospitalization. The febrile questionnaire included data regarding febrile episodes, symptoms, dates, duration, severity, movements outside the area, utilization of health services, contacts and blood sample results (serology).

Symptomatic dengue was detected through passive surveillance for dengue-associated symptoms and absenteeism from schools. The clinical team of FSD visited schools every week, checked attendance, and visited the homes of absentees. If absentees had a febrile illness in the previous week, they were evaluated with a physical exam and an acute blood sample to confirm infection. In addition, this population was monitored for febrile symptoms and suspected dengue illness through a toll-free number where parents could call for medical assistance when febrile disease appeared in any member of the family. This free of charge telephone line was directed only to the members of the cohort so as to voluntarily report febrile cases to the team (FSD) where a physician in charge would respond to their request.

Participants were defined as lost to follow up after a full year had passed since their previous blood sample, despite repeated attempts to locate the participant, or if there was a verifiable reason for dropping from the study (voluntary request from the participant, movement from the study area).

### Laboratory procedures

Baseline serum samples were taken to test for IgG antibodies by capture enzyme-linked immunosorbent assay (ELISA-Panbio). A 5ml of peripheral venous blood was obtained with BD Vacutainer and centrifuged at 3000 rpm (Bio-Lion XC-L4). The serum was stored at 4±2°C and aliquots obtained with Scilogex micropette plus autoclavable pipettor of 500μl at -70°C (Eppendorf CryoCube F570-86 Upright). Following the reference values, negative, equivocal and positive results were determined as <9, 9–11 and >11 Panbio units, respectively.

Febrile episodes in enhanced surveillance were classified as DENV infections based on NS1 and IgM serology.

### Statistical analysis

Descriptive analysis of the cohort included school children and their siblings aged 0 to 15 years. Participants were stratified in three age groups (0 to 5, 6 to 8 and 9 to 15 years old). Dengue IgM and IgG results above >11 Panbio units were considered positive for dengue and were included as the dependent variable in the analysis. The logistic regression analysis was adjusted by age and sex and was used to identify risk factors for dengue infection. Odds ratios (ORs) and their 95% confidence intervals were calculated and statistically significant differences (p<0.05) were included in the final model. The analysis was done with STATA 14.2.

The protocol was approved by the Ethics and Research Committee from the O´Horan General Hospital from the state Ministry of Health, Register No. CEI-0-34-1-14 and the review board at the Fred Hutchinson Cancer Research Center.

## Results

**Fig 1:**
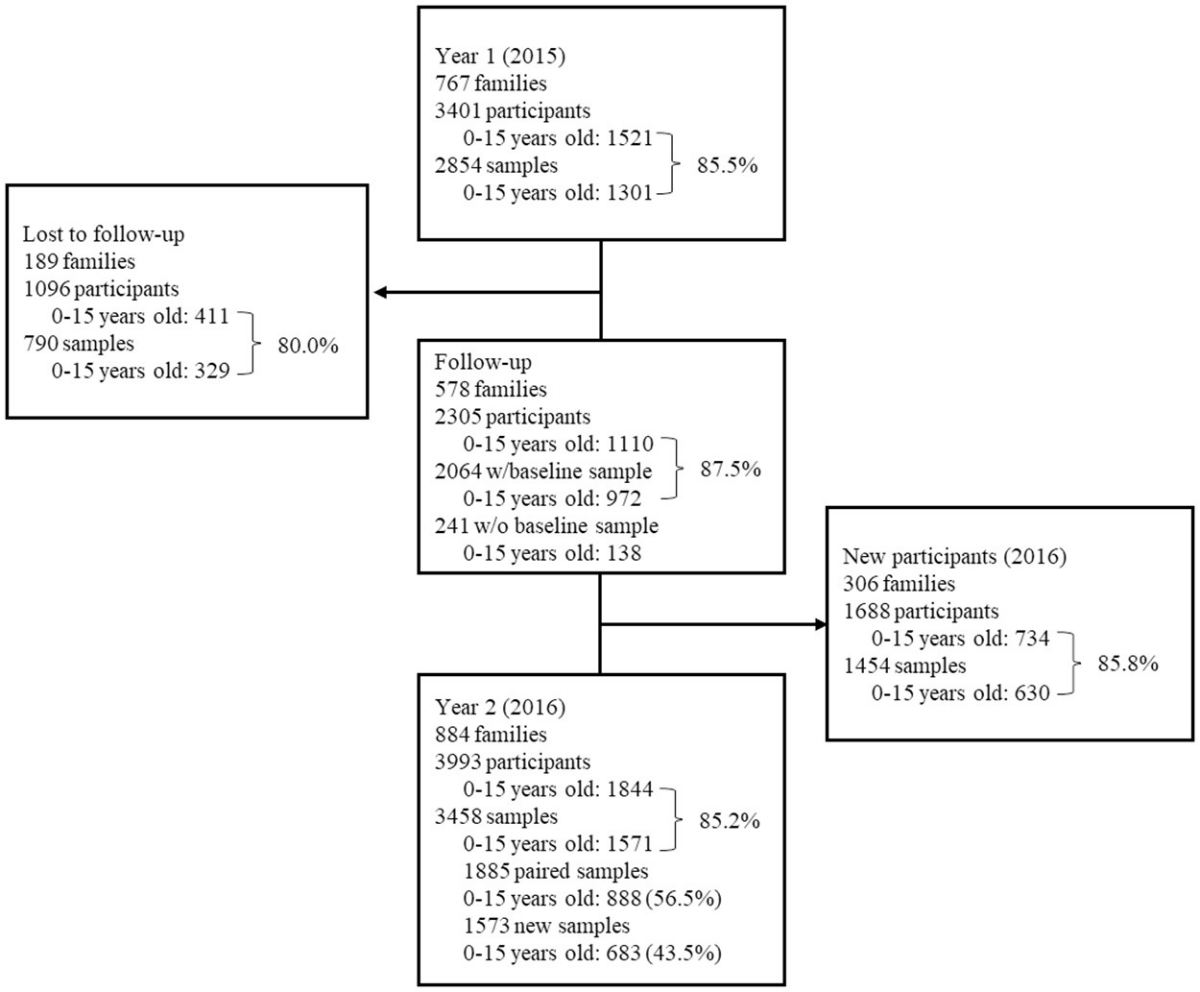
Cohort of families and school children aged 0 to 15 years old, 2015–2016. Percent represents blood samples coverage of 0 to 15 years old.

A total of 767 index children and 3,401 family members were recruited in year 1. In Merida, 463 families and index school children and 448 siblings aged 0 to 15 years old were enrolled at baseline from eight elementary schools: 151 from low risk area, 153 from middle risk and 159 from high risk area. In Ticul, a total of 151 families and index school children were recruited from two elementary schools along with 170 siblings aged 0 to 15 years old. In Progreso, 153 families and index school children from four elementary schools together with 136 siblings were selected (Table 1). The total number of children aged 0 to 15 years old in the 2015 cohort was 1,521 of whom 1301 (85%) had blood sampled. During the period we lost 189 families that add up to 1,096 individuals, 37% of which (411) were under 15 years old. The baseline cohort (year 1) ended with 578 families and 2,305 individuals of whom 1,110 (48.2%) were children aged 0 to 15 years old. Baseline samples for this first year accounted for 972 (87%) of school children in the cohort. The enrolment of new index children (middle plus elementary schools) incorporated 306 new families and index students along with 1,688 new participants, including 734 new children aged 0 to 15 years old (Fig 1).

**Table 1:** Cohort of school children by city, Yucatan, 2015 to 2016.

2015ʹs Cohort (Y1)		Merida		Progreso		Ticul		Total	
		N	(%)	N	(%)	N	(%)	N	(%)
Recruitment	Index children	463	51	153	53	151	47	767	50
	Siblings	448	49	136	48	170	53	754	50
	**Total**	**911**	**100**	**289**	**100**	**321**	**100**	**1521**	**100**
Lost to follow up	Index children	149	32	21	14	19	13	189	25
	Siblings	155	35	23	17	44	26	222	29
	**Subtotal**	**304**	**33**	**44**	**15**	**63**	**20**	**411**	**27**
Follow up	Index children	314	68	132	86	132	87	578	75
	Siblings*	347	65	147	83	134	74	628	71
Baseline cohort (Y1)	**Total**	**661**	**100**	**279**	**100**	**266**	**100**	**1206**	**100**
New participants (2016)	Index children	171	48	66	47	69	48	306	48
	Siblings	185	52	73	53	74	52	332	52
	**Subtotal**	**356**	**100**	**139**	**100**	**143**	**100**	**638**	**100**
**Cohort 2016 (Y2)**	Index children	485	48	198	47	201	49	884	48
	Siblings	532	52	220	53	208	51	960	52
**Total cohort**		**1017**	**100**	**418**	**100**	**409**	**100**	**1844**	**100**

*Total includes siblings not participating in Y1 but decided to participate in Y2.

The baseline cohort experienced losses to follow-up and drop out in different proportions in the five areas studied. Of the total 2,255 school children recruited in both years we included in this report 1,844 children aged 0 to 15 years (884 index children) with a total of 1,571 blood samples taken (85%) (S1 Table). The results of paired samples taken in the same individuals in baseline and follow up are presented elsewhere.

Voluntary dropouts were more frequent in Merida (21.8%) than in Progreso (9.8%) or Ticul (9.9%). Dropout due to the family moving from the area of study or not found in subsequent visits was much higher in Merida (10.4%) than Progreso (3.9%) or Ticul (2.6%). We had a 76% success of follow up in Merida but higher in Progreso (90%) and Ticul (91%) (Table 1). The family patterns are basically nuclear with similar composition between cities, nevertheless, families lost to follow-up had a higher average number of individuals per family (5.9, range 2–9) than families that were followed up (4.8, range 2–10). Progreso and Ticul showed more stable patterns of mobility within the neighborhoods where they lived, which was not the case in Merida.

### Demographic characteristics of the cohort

Sex distribution in the cohort of school children was very similar (48.7% women and 51.3% men) in all risk areas although low risk Merida had 59.1% male children. The age distribution of all children was 14.3% for children under five years old, 39.9% for 6 to 8 years old and 45.9% for children aged 9 to 15 years old. Ticul had the lowest proportion of children under five years (8.6%) (Table 2).

**Table 2:** Demographic characteristics of school children in Yucatán, 2016.

Variables	Merida						Progreso n (%)		Ticul n (%)		Total n (%)	
	LR n (%)		MR n (%)		HR n (%)							
Sex												
Women	124	(40.9)	180	(47.9)	169	(50.0)	207	(49.5)	218	(53.3)	898	(48.7)
Men	179	(59.1)	196	(52.1)	169	(50.0)	211	(50.5)	191	(46.7)	946	(51.3)
Total	303	(100)	376	(100)	338	(100)	418	(100)	409	(100)	1844	(100)
Age												
0–5	46	(15.2)	62	(16.5)	50	(14.8)	70	(16.7)	35	(8.6)	263	(14.3)
6–8	120	(39.6)	141	(37.5)	125	(37.0)	169	(40.4)	180	(44.0)	735	(39.9)
9–15	137	(45.2)	173	(46.0)	163	(48.2)	179	(42.8)	194	(47.4)	846	(45.9)
Total	303	(100)	376	(100)	338	(100)	418	(100)	409	(100)	1844	(100)

LR: Low risk, MR: Medium risk, HR: high risk.

### Socioeconomic conditions of households

House property patterns in Yucatan showed that most of the houses were privately owned (70.5%), few were rented (4.5%) and 25% shared with other families (usually kin). Construction materials of walls, floor and roof were cement (>95%) although palm roofs in Progreso (7.7%) and Ticul (7.9%) described particular socioeconomic conditions of some families participating in the study. Most of the houses (72%) had 2 to 3 rooms while 18.8% of houses in Ticul, low risk Merida (18.2%) and high risk Merida (15.9%) had 4 to 5 rooms. Protection with screens in doors and windows was a common practice in this region with almost 95% of houses with at least one door or window protected. The presence of kitchen and bathroom outside the house was much more common in Ticul (25.7% and 24.1% respectively) than in Progreso (3.3% and 12.6%) and the high risk area in Merida (7.7% and 12.6%). Access to potable water was also a common feature (>90%) in the three cities but the need to store water for several domestic activities was also very common (45.9%) in all sites, particularly in Ticul that reported a higher need to store potable water (78%). Garbage collection was a widespread public service in the areas, although in Ticul, 19.4% of households reported the need to burn or throw away the garbage (Table 3).

**Table 3:** Households characteristics in Merida, Ticul and Progreso, 2016.

Variables	Merida						Progreso n (%)		Ticul n (%)		Total n (%)	
	LR n (%)		MR n (%)		HR n (%)							
House												
Own	124	(75.2)	130	(79.3)	127	(69.8)	143	(78.6)	99	(51.8)	623	(70.5)
Rent	8	(4.8)	8	(4.9)	11	(6)	13	(7.1)			40	(4.5)
Shared	33	(20)	26	(15.9)	44	(24.2)	26	(14.3)	92	(48.2)	221	(25)
Total	165	(100)	164	(100)	182	(100)	182	(100)	191	(100)	884	(100)
Roof												
Cement	165	(100)	164	(100)	180	(98.9)	168	(92.3)	176	(92.1)	853	(96.5)
Palm					2	(1.1)	14	(7.7)	15	(7.9)	31	(3.5)
Total	165	(100)	164	(100)	182	(100)	182	(100)	191	(100)	884	(100)
Floor												
Cement	165	(100)	164	(100)	182	(100)	181	(99.5)	188	(98.4)	880	(99.5)
Total	165	(100)	164	(100)	182	(100)	182	(100)	191	(100)	884	(100)
Walls												
Cement	165	(100)	164	(100)	181	(99.5)	179	(98.4)	186	(97.4)	875	(99)
Wood					1	(0.5)	3	(1.6)	5	(2.6)	9	(1)
Total	165	(100)	164	(100)	182	(100)	182	(100)	191	(100)	884	(100)
Rooms												
1	7	(4.2)	2	(1.2)	20	(11)	39	(21.4)	24	(12.6)	92	(10.4)
2 to 3	124	(75.2)	136	(82.9)	125	(68.7)	129	(70.9)	123	(64.4)	637	(72.1)
4 to 5	30	(18.2)	22	(13.4)	29	(15.9)	13	(7.1)	36	(18.8)	130	(14.7)
>5	4	(2.4)	4	(2.4)	8	(4.4)	1	(0.5)	8	(4.2)	25	(2.8)
Total	165	(100)	164	(100)	182	(100)	182	(100)	191	(100)	884	(100)
Screened windows												
0	10	(6.1)	2	(1.2)	10	(5.5)	9	(4.9)	9	(4.7)	40	(4.5)
1	47	(28.5)	59	(36)	51	(28)	99	(54.4)	28	(14.7)	284	(32.1)
2	62	(37.6)	73	(44.5)	77	(42.3)	58	(31.9)	124	(64.9)	394	(44.6)
3 or more	46	(27.9)	30	(18.3)	44	(24.2)	16	(8.8)	30	(15.7)	166	(18.8)
Total	165	(100)	164	(100)	182	(100)	182	(100)	191	(100)	884	(100)
Screened doors												
0	3	(1.9)	1	(0.6)	3	(1.7)	4	(2.2)			11	(1.3)
1	22	(13.9)	32	(19.9)	29	(16.4)	42	(23.1)	10	(5.2)	135	(15.5)
2	93	(58.9)	105	(65.2)	127	(71.8)	119	(65.4)	166	(86.9)	610	(70.2)
3 or more	40	(25.3)	23	(14.3)	18	(10.2)	17	(9.3)	15	(7.9)	113	(13)
Total	158	(100)	161	(100)	177	(100)	182	(100)	191	(100)	869	(100)
Kitchen												
Inside	160	(97)	162	(98.8)	167	(92.3)	176	(96.7)	142	(74.3)	807	(91.4)
Outdoors	5	(3)	2	(1.2)	14	(7.7)	6	(3.3)	49	(25.7)	76	(8.6)
Total	165	(100)	164	(100)	181	(100)	182	(100)	191	(100)	883	(100)
Bathroom												
WC	165	(100)	164	(100)	177	(97.3)	171	(94)	183	(95.8)	860	(97.3)
Letrine/outdoors					5	(2.7)	11	(6)	8	(4.2)	24	(2.7)
Total	165	(100)	164	(100)	182	(100)	182	(100)	191	(100)	884	(100)
Bathroom												
Inside	160	(97)	161	(98.2)	159	(87.4)	159	(87.4)	145	(75.9)	784	(88.7)
Outdoors	5	(3)	3	(1.8)	23	(12.6)	23	(12.6)	46	(24.1)	100	(11.3)
Total	165	(100)	164	(100)	182	(100)	182	(100)	191	(100)	884	(100)
Potable water												
Yes	146	(88.5)	159	(97)	173	(95.1)	171	(94.0)	189	(99)	838	(94.8)
Total	165	(100)	164	(100)	182	(100)	182	(100)	191	(100)	884	(100)
Storing water												
Yes	60	(36.4)	75	(45.7)	86	(47.3)	36	(19.8)	149	(78)	406	(45.9)
No	105	(63.6)	89	(54.3)	96	(52.7)	146	(80.2)	42	(22)	478	(54.1)
Total	165	(100)	164	(100)	182	(100)	182	(100)	191	(100)	884	(100)
Garbage												
Municipal service	164	(99.4)	163	(99.4)	178	(97.8)	166	(91.2)	154	(80.6)	825	(93.3)
Burns/throw	1	(0.6)	1	(0.6)	4	(2.1)	16	(8.7)	37	(19.4)	59	(7.7)
Total	165	(100)	164	(100)	182	(100)	182	(100)	191	(100)	884	(100)

LR: Low risk, MR: Medium risk, HR: high risk.

### Serology results

The percentage of blood sampling in 1,521 children in the 2015 cohort and 1,844 children in 2016 was 85% (Fig 1). Ticul had the highest coverage (90.5%) of blood samples, Merida had similar coverages by risk areas (87%), while Progreso presented the lowest coverage (75.6%). There were 888 (68.3%) paired samples in children. Again, Ticul led the blood sampling with 79.3%, followed by Progreso (70%) and Mérida (63.7%). (S1 Table).

**Fig 2:**
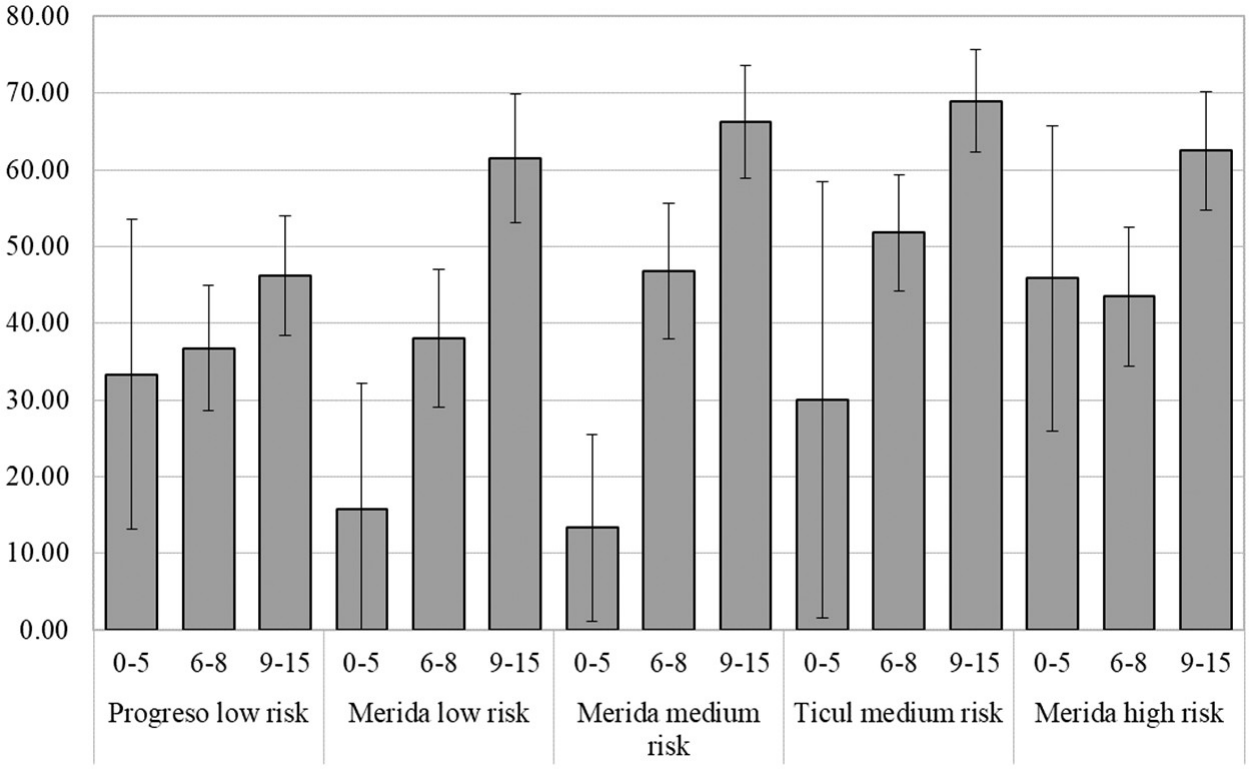
Seroprevalence of school children by age group and risk areas, Yucatan, 2016. Seroprevalence is represented by percentage and 95% CI.

Seroprevalence of 0 to 15 years old was 46.8 (CI 95% 44.1–49.6). Overall seroprevalence to dengue showed no difference in females (53.9%, 95% CI: 50.4–57.4) compared to males (49.4%, 95% CI: 45.8–52.9) and differences by age group or city were not significant except in Ticul (61%, 95% CI: 53.9–67.9) for female vs male (49.4% 95% CI: 45.82–52.87). Seroprevalence increased with age and was significantly lower in 0 to 5 years old (26.9%, 95% CI:18.4–35.4) compared with children 6 to 8 years old (43.9%, 95% CI:40.1–47.7) and 9 to 15 years old (61.4%, 95% CI:58.0–64.8). Seroprevalence by age group and risk area showed significant differences between the 0 to 5 years old compared to the 9 to 15 years old in low risk Merida (15.8%, 95% CI: -0.61–32.2 vs 61.5%, 95% CI:53.2–69.9), medium risk Merida (13.3%, 95% CI:1.2–25.5 vs 66.2%, 95% CI:58.9–73.6) and Ticul (30%, 95% CI:1.6–58.4 vs 69.0%, 95% CI:62.3–75.6) but not in Progreso or high risk Merida. Differences between 6 to 8 years old and the 9 to 15 age groups were significant in all risk areas except Progreso (Fig 2).

### Seroprevalence by schools

**Fig 3:**
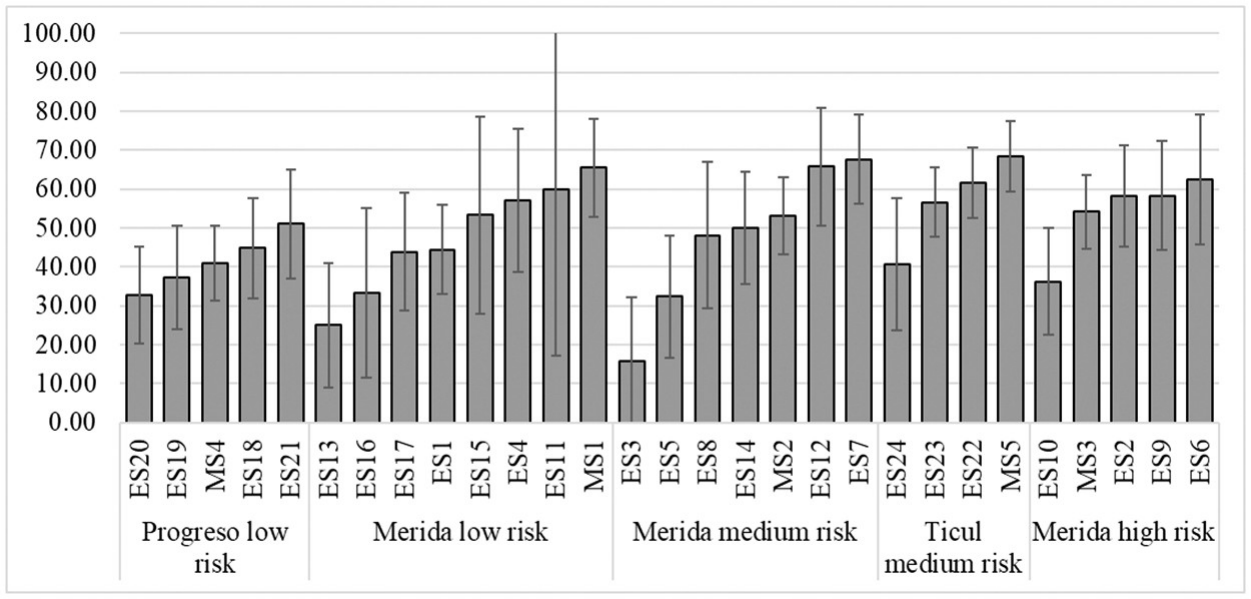
Seroprevalence by schools and risk areas, Yucatan, 2016. Seroprevalence is represented by percentage and 95% CI.

Seroprevalence by school showed variations in dengue exposure within areas of risks. The lowest rate in low risk areas like Progreso or low risk area in Merida are not different from the lowest rate in Ticul or high risk Merida. Similar patterns appeared in the schools with the highest seroprevalence in each risk area (Fig 3).

### Risk factors and dengue infection

The multivariate analysis of children with blood results showed no difference by sex but dengue risk increased with age, being four times higher for those 9 to 15 years old compared to children under five years old. While parents reported very few cases of dengue in the previous year, the history of dengue was significant as well as the report of dengue confirmation by health personnel. Regarding the risks derived from household conditions obtained by the questionnaires: sharing the domestic space with other families increased the risk 1.7 times over the individual families that own or rented their house, and the risk of dengue was significantly higher when kitchen and bathroom were located outside the house. Protection with screens of windows showed a good level of protection only when screens cover all the windows. While bivariate analysis of the prevalence of dengue by urban areas showed discrete differences, the analysis combining areas of risk (Progreso and low risk Merida vs. medium risk Merida and Ticul, and high risk Merida alone) pointed out that risk of infection was significantly higher in the medium and high risk areas compared to those in the low level risk (Table 4).

**Table 4:** Risk factors and dengue infection school children 0 to 15 years old, Yucatan, 2016.

Factors	OR	CI 95%	p value (<0.05)*
Sex			
Woman	1.0		
Man	0.9	0.8–1.1	0.370
Age*			
0 to 5	1.0		
6 to 8	1.9	1.3–3.0	0.002*
9 to 15	4.1	2.7–6.3	0.000*
History of dengue			
No	1.0		
Yes	4.3	1.8–10.1	0.001*
Confirmed by health personnel			
No	1.0		
Yes	7.1	2.1–24.5	0.002*
House property			
Rent	1.0		
Own	0.7	0.3–1.3	0.334
Shared	1.7	1.2–2.3	0.001*
Windows with screens			
0	1.0		
1–2	0.6	0.3–1.2	0.15
3 or more	0.4	0.2–0.9	0.034*
Doors with screens			
0–1	1.0		
2–3	1.8	1.2–2.7	0.001*
Kitchen			
Inside	1.0		
Outside	1.9	1.2–3.1	0.005*
Bathroom			
Inside	1.0		
Outside	1.8	1.2–2.8	0.004*
Transmission risk			
Low/Progreso	1.0		
Medium/Ticul	1.6	1.3–2.1	0.000*
High	1.4	1.1–1.9	0.005*

*Age is adjusted by sex, all other variables adjusted by age and sex.

### School absenteeism

Surveillance of absenteeism in the schools showed 98 cases of absentees due to fever (January to June 2015). An outbreak of 57 cases of chickenpox was detected along with three likely cases of dengue that had negative test results. During the second semester, an outbreak of chikungunya emerged in the State of Yucatan, four schools closed for four days and 20 probable cases of dengue and 34 of chikungunya were detected. In Ticul, 44 children were detected as absentees and only three probable cases of dengue were detected with negative results. A total of seven cases of chikungunya were reported. In Progreso, 11 children were absent, six cases were respiratory infections, two diarrheas, two cases of conjunctivitis and one with chickenpox. From September to November, three fever cases were reported, two suspected cases of dengue that were negative. During 2016, absenteeism surveillance reported 434 total cases of absenteeism, 219 cases (50%) due to fever. Four cases were surgical pathologies and only two clinical dengue cases; 107 cases of respiratory diseases and 78 chickenpox cases (46% and 51% were in Merida and Ticul schools), 28 cases were not located. A total of 53 cases of absenteeism were reported in cohort students: 23% (12/53) were non-febrile cases; 77% due to febrile pathologies such as allergy symptoms (1), abdominal pain (1), respiratory tract infections (27), urinary infection (1), chickenpox (10) and one family was not located.

### Toll-free number

During 2015, 373 telephone calls were received by the FSD group, 32.5% (n = 121) were not related to a febrile episode and patients were referred to their family physician or clinic. Around 59.1% (n = 149/252) of febrile patients that contact the dengue line had previously consulted a physician who gave the diagnosis of probable dengue and people requested the blood sample for confirmation of clinical diagnosis. The febrile patients (n = 103/252) who did not consult a physician, 59.2% (n = 61/103) mentioned previous contact with a dengue case in their neighborhood. Of all febrile patients that contact the dengue line, 78.82% (n = 294) had 2 or more days with fever (>38 °C). The age group that more frequently contact the FSD line was the 20–49 years group (41.5%), followed by the 5–9 years old (25%), >50 years (13.3%), 10–14 years (10.2%), 15–19 years (5.5%) and 0–4 years old (4.5%). The average number of days between the beginning of the fever and the blood sample was 3.9±3.7 days, with significant variations between cities: Merida 4.4 days, Progreso 3.2 days and Ticul 2.8 days.

Through the enhanced surveillance strategies (absenteeism and toll-free number) 244 serological tests for dengue (IgM or NS1) were performed, 59% (n = 144) from Merida, 17% (n = 42) from Progreso and 24% (n = 58) from Ticul. Only 8% of these samples were positive for dengue.

## Discussion

The results provided by the cohort of school children in three urban settings in the state of Yucatan, Mexico, described the high exposure to dengue infection in scholar groups that increased with age, without differences by sex but with significant differences between low, medium and high risk areas. The prevalence of antibodies in children 9 to 15 years above 60% in all areas except Progreso (low risk) confirmed the high transmission of dengue virus in this endemic state of the country.

Prevalence found in this study were as expected and comparable to those from other endemic countries inside and outside the Americas, as reported in urban Nicaragua (2003) [24,25] or in urban settings in Central Brazil (2001) and northeast Brazil (2013) [26] with similar exposure and prevalence rates. Studies in India also showed increasing dengue infection with age and differences by region [27] were comparable to those reported in other endemic countries like Indonesia (1996) [28]; Sri Lanka (2014) [29] and Vietnam [30]. In the case of Yucatan, a state level prevalence of 72.5% was reported since 1985 [31]; seroprevalences in school children 8 to 14 years old (1987–1988) in urban (56.8%) and rural Merida (63.7%) [32] demonstrated the high exposure to dengue in the past, while another serological surveys done in Yucatan in 1996 and 2006 demonstrated seroprevalences of 22% and 20% in under five years old and 30% to 51% in 5 to 14 years old, respectively [33]. In other states of the country similar prevalence have been also reported: 35.7% in 5 to 9 years old and 52.2% for 10 to 14 years old (2011) [34].

Dengue transmission is highly heterogeneous and the burden varies by geographic region [35], countries [36], age groups affected and serotype [37]. Significant heterogeneity in transmission intensity has been identified within districts, sub-districts and even finer spatial scales like schools [38–41]. The differences between risk areas was expected since social, economic and environmental conditions have all proven to have certain influence in infection risks of populations [42,43]. These conditions could also influence transmission within the different schools in the selected areas. The low risk areas selected in our study comprise one urban area in the capital city and a town (Progreso) with historical low and occasional reports of dengue cases. Overall seroprevalence in these sites was significantly lower than the reported for medium and high risk areas. The higher prevalence of dengue infection in all age groups in Ticul (medium risk) demonstrated that conditions for transmission were wider than expected. The under report of cases by local health services as well as less demand for care from the sick population could be some distinctive traits within this community. Population movements between Ticul and high risk areas of Merida due to economic dynamics could also be a contributing factor. On the other hand, we did identify certain household risk conditions (storage of water [44], bathroom and kitchen outdoors) in Ticul that could help explain this particular situation. Window screening has been suggested as an important feature to prevent *Aedes* from entering houses in Merida and potentially affect dengue transmission risk as well [45]. In this study, having ample coverage of screens in windows and doors was protective and urban programs should promote the inclusion of this preventive measure in houses in high risk areas [46,47].

Under reported cases is also a very common trait in school children in several settings [48,49]. The long history of dengue in Yucatan has created conditions where dengue is no longer identified as a health threat; fever is confused with other diseases and appears as a nonspecific febrile syndrome. The enhanced strategies introduced (school absenteeism and toll-free number) helped identify new cases within the school cohort although coverage and use of the telephone line needs to be promoted within the community to become a reliable and useful surveillance tool. Regular community home-to-home visits have proven to be an additional and useful strategy to support the traditional surveillance established by the health sector [50].

Circulation of DEN-1, DEN2 and DEN-4 serotypes in the Yucatan region has not changed although the recent introduction of DEN-3 virus in 2016 could increase transmission due to low herd immunity towards this serotype. The introduction of chikungunya virus in 2015 and Zika virus in 2016 competed with all dengue serotypes and diminished the capacity to identify dengue cases by health providers and family members in the cohort as well. The enhance surveillance strategies implemented help improve detection of dengue cases under this circumstance.

Limitations in our cohort are linked to the problems arising from the difficulties to engage family members and individuals in this kind of study. Losses to follow-up were higher in Merida and drop-out families were larger than those who stayed participating and it could generate under estimations of the risk of dengue in this city. The coincidence of chikungunya and Zika epidemics virus produced competing conditions for diagnosis and promoted intensive control interventions that eventually diminished our capacity to identify dengue cases or lower the transmission of dengue infection in the community. Nevertheless, the enhanced surveillance strategies allowed us to identify the three infections in our cohort. We did not perform entomological surveys to establish potential differences in vector densities by risk areas and these data could provide additional to support our findings. Screening of doors and windows may behave as a proxy of entomological risk since areas with this kind of protection are prone to have higher mosquito densities.

With the potential introduction of innovative preventive or vector control interventions at sight, it is imperative that countries improve their surveillance system and produce baseline data that describe the epidemiological profile of the target population in order to improve the estimates of the direct and indirect effects of these individual or combined interventions. Our results confirmed the high exposure in these age groups and provide evidence that preventive and control interventions directed to children could decrease the burden of disease in high transmission areas [51].

## Supporting information

S1 Table Blood sampling in schoolchildren (baseline, follow-up and new members, 2015–2016). (DOCX)

S2 Table STROBE Statement-checklist of items that should be included in reports of observational studies. (DOC)
