# Supplementary material for: Dengue seroprevalence in a cohort of schoolchildren and their siblings in Yucatan, Mexico (2015-2016)
Source: PLoS Negl Trop Dis. 2018 Nov 21;12(11):e0006748. doi: 10.1371/journal.pntd.0006748 (PMC6248890; doi:10.1371/journal.pntd.0006748)
Supplement: S1 Table — (DOCX) [file pntd.0006748.s001.docx]

**S1 Table. Blood sampling in schoolchildren (baseline, follow-up and new members, 2015-2016)**

| **Age groups per area** | **2015** | **Samples 2015** | | **Single samples 2015** | | **Paired samples 2015** | | **2016** | **New samples 2016** | | **Samples 2016**  **(paired 2015 + new 2016)** | |
| --- | --- | --- | --- | --- | --- | --- | --- | --- | --- | --- | --- | --- |
|  | **N** | **n** | **%** | **n** | **%** | **n** | **%** | **N** | **n** | **%** | **n** | **%** |
| **Merida** | 911 | 774 | 85 | 493 | 63.7 | 281 | 36.3 | 1017 | 392 | 44.3 | 885 | 87 |
| ***Low*** | 297 | 246 | 82.8 | 173 | 70.3 | 73 | 29.7 | 303 | 90 | 34.2 | 263 | 86.8 |
| 0-5 | 43 | 14 | 32.6 | 8 | 57.1 | 6 | 42.9 | 46 | 11 | 57.9 | 19 | 41.3 |
| 6 to 8 | 149 | 139 | 93.3 | 93 | 66.9 | 46 | 33.1 | 120 | 21 | 18.4 | 114 | 95 |
| 9 to 15 | 105 | 93 | 88.6 | 72 | 77.4 | 21 | 22.6 | 137 | 58 | 44.6 | 130 | 94.9 |
| ***Medium*** | 292 | 249 | 85.3 | 159 | 63.9 | 90 | 36.1 | 376 | 166 | 51.1 | 325 | 86.4 |
| 0-5 | 38 | 15 | 39.5 | 6 | 40 | 9 | 60 | 62 | 25 | 80.6 | 31 | 50 |
| 6 to 8 | 165 | 160 | 97 | 98 | 61.3 | 62 | 38.8 | 141 | 31 | 24 | 129 | 91.5 |
| 9 to 15 | 89 | 74 | 83.1 | 55 | 74.3 | 19 | 25.7 | 173 | 110 | 66.7 | 165 | 95.4 |
| ***High*** | 322 | 279 | 86.6 | 161 | 57.7 | 118 | 42.3 | 338 | 136 | 45.8 | 297 | 87.9 |
| 0-5 | 43 | 16 | 37.2 | 9 | 56.3 | 7 | 43.8 | 50 | 15 | 62.5 | 24 | 48 |
| 6 to 8 | 161 | 153 | 95 | 95 | 62.1 | 58 | 37.9 | 125 | 23 | 19.5 | 118 | 94.4 |
| 9 to 15 | 118 | 110 | 93.2 | 57 | 51.8 | 53 | 48.2 | 163 | 98 | 63.2 | 155 | 95.1 |
| **Progreso** | 289 | 247 | 85.5 | 173 | 70 | 74 | 30 | 418 | 143 | 45.3 | 316 | 75.6 |
| 0-5 | 37 | 7 | 18.9 | 4 | 57.1 | 3 | 42.9 | 70 | 17 | 81 | 21 | 30 |
| 6 to 8 | 152 | 146 | 96.1 | 107 | 73.3 | 39 | 26.7 | 169 | 31 | 22.5 | 138 | 81.7 |
| 9 to 15 | 100 | 94 | 94 | 62 | 66 | 32 | 34 | 179 | 95 | 60.5 | 157 | 87.7 |
| **Ticul** | 321 | 280 | 87.2 | 222 | 79.3 | 58 | 20.7 | 409 | 148 | 40 | 370 | 90.5 |
| 0-5 | 39 | 10 | 25.6 | 3 | 30 | 7 | 70 | 35 | 7 | 70 | 10 | 28.6 |
| 6 to 8 | 165 | 162 | 98.2 | 133 | 82.1 | 29 | 17.9 | 180 | 37 | 21.8 | 170 | 94.4 |
| 9 to 15 | 117 | 108 | 92.3 | 86 | 79.6 | 22 | 20.4 | 194 | 104 | 54.7 | 190 | 97.9 |
| **Total** | 1521 | 1301 | 85.5 | 888 | 68.3 | 413 | 31.7 | 1844 | 683 | 43.5 | 1571 | 85.2 |
