# Supplementary material for: Dengue seroprevalence in a cohort of schoolchildren and their siblings in Yucatan, Mexico (2015-2016)
Source: PLoS Negl Trop Dis. 2018 Nov 21;12(11):e0006748. doi: 10.1371/journal.pntd.0006748 (PMC6248890; doi:10.1371/journal.pntd.0006748)
Supplement: S2 Table — (DOC) [file pntd.0006748.s002.doc]

**S2 Table. STROBE Statement-checklist of items that should be included in reports of observational studies**

|  | Item No | Recommendation |
| --- | --- | --- |
| **Title and abstract** | 1 | (*a*) Both tittle and abstract indicate not only the design (cohort) and period of the study (2015-2016), but also provide information of the participants (schoolchildren and their siblings in Yucatan). |
|  |  | (*b*) The abstract also specifies the age range (0-15 years) and the objective (provide baseline epidemiological information to evaluate the direct and indirect effects of vector control interventions and new tools, as the potential introduction of dengue vaccine population, methods and main results). |
| Introduction | | |
| Background/rationale | 2 | Background and rationale for the investigation were reported clearly and concisely. |
| Objectives | 3 | Objectives were presented immediately after the introduction, and there were no study hypotheses |
| Methods | | |
| Study design | 4 | Key elements of study design were presented early in the paper (in the abstract, introduction and methods) and organized by sections of the methodology. |
| Setting | 5 | We describe the characteristics of the study sites and their population, enrolment and follow up periods, including all the procedures linked to data collection. |
| Participants | 6 | We mentioned the eligibility criteria and described the sources and methods of selection of participants and follow-up. Lost to follow up were defined in the paper. |
| Variables | 7 | Outcomes, exposures and diagnosis criteria were clearly specified, and the potential confounders were discussed when applied. |
| Data sources/ measurement | 8 | Variable related to the household characteristics were not defined because of we considered that its concept was clear and did not required it. Sample collection, laboratory criteria and its procedures were fully described. |
| Bias | 9 | The authors declared not to have any interested conflict and samples were repeated if equivocal results were obtained. |
| Study size | 10 | The study size was determined by convenience and declared in the manuscript. |
| Quantitative variables | 11 | Management of quantitative variables is explained in the statistical analysis section. Only participants age was stratified (in three age groups) and it was mentioned. |
| Statistical methods | 12 | All statistical methods (descriptive statistics, logistic regression and odds ratio) were specified, including the adjustment by age and sex (in logistic regression) to identify risk factors. Confidence intervals and significance level were also stablished. Missing data and lost to follow up are reflected in tables or supportive information and were eliminated for the analysis. |

| Results | |  |
| --- | --- | --- |
| Participants | 13 | The number of individuals at each stage of study is presented in a flow diagram and contained in informative tables. Some numbers (as potentially eligible and examined for eligibility) were not included in the manuscript due to the editing process. Reasons for loss of follow up are also presented in a table. |
| Descriptive data | 14 | Relevant characteristics of the participants and its households are all included in the manuscript. |
|  |  | We present missing data by providing the proportion of follow-up by total and subtotal numbers in all the tables to clarify as possible the data. |
|  |  | Follow-up time and participants were summarised by stages and proportions. |
| Outcome data | 15 | Numbers by outcome events (i.e. baseline, follow up and acute samples) are reported by sections and objectives. |
| Main results | 16 | The results were described by section and indicated adjustment when applied. All estimates as precision and significance level were presented. |
| Other analyses | 17 | Don’t apply. |
| Discussion | |  |
| Key results | 18 | Key results were presented according to the study objectives and discussed considering the limitations of the study. |
| Limitations | 19 | The limitations of the study were mainly related to the study size, and the complexity to maintain a cohort of this characteristics. Limitations were considered and discussed throughout the manuscript. |
| Interpretation | 20 | The results were interpreted considering the objectives, limitations, and characteristics of the analyses and compared with similar studies to established objective conclusions. |
| Generalisability | 21 | Due to heterogeneity of dengue transmission, our findings may be considered valid in areas and population with similar characteristics to our cohort. |
| Other information | |  |
| Funding | 22 | Sanofi-Pasteur financed this Project (DNG25) but did not intervene in the design, study development, collection, management, information analysis or presentation of results. This project was also supported by National Institutes of Health Grants  R37 AI032042 and U54 GM111274. Authors declare not conflict of interest. |
